# Validation of MEDFICTS Dietary Assessment Questionnaire in Turkish Population

**DOI:** 10.1017/S1368980021002299

**Published:** 2022-01

**Authors:** Zeynep Göktaş, Derya Dikmen, Neslişah Rakıcıoğlu

**Affiliations:** Hacettepe University, Faculty of Health Sciences, Department of Nutrition and Dietetics, Ankara 06100, Turkey

**Keywords:** MEDFICTS, Validation, Dietary fat intake

## Abstract

**Objective:**

The purpose of this study was to assess the validity of MEDFICTS (Meats, Eggs, Dairy, Fried foods, fat In baked goods, Convenience foods, fats added at the Table, Snacks) questionnaire in Turkish population.

**Design:**

MEDFICTS questionnaire is a brief dietary assessment tool developed as part of the National Cholesterol Education Program Adult Treatment Panel guidelines, and it measures the adherence to Step 1 and Step 2 diets that are recommended for the prevention and treatment of CVD. MEDFICTS questionnaire was administered with 3-d food record to compare overall dietary fat intake.

**Setting:**

This study was conducted at the Hacettepe University (Ankara, Turkey) in 2017.

**Participants:**

Subjects were university students, recruited from several departments of Hacettepe University by trained dietitians. A total of 442 adults (249 females and 194 males) between the ages of 18 and 31 years participated in the study. Students with CVD were excluded.

**Results:**

Total fat intake ratio was higher than the recommended level for both males and females (39·4 % and 39·9 %, respectively). Mean MEDCISTS score was 66·3 ± 27·24 points. Total energy, total fat, SFA and cholesterol intakes from 3-d food records within the different MEDFICTS diet groups significantly differed (*P* < 0·001 for all). Receiver operating characteristics curve analysis demonstrated that a cut-off point of 60 showed 80 % sensitivity and 65 % specificity.

**Conclusions:**

Our data indicate that the MEDFICTS questionnaire is moderately accurate; however, sensitivity analysis did not demonstrate the recommended 40 points as an optimal cut-off point for Turkish population.

CVD is still the number one cause of death in developed countries despite the declining trend of global burden of CVD in the last decade^(1,2)^. CVD is the cause of one of every three deaths in the USA and one of every four deaths in Europe^(2,3)^. Furthermore, in developing countries, the incidence of CVD dramatically increased in the last 25 years due to the transition to Western diet in these countries^(4,5)^. In Turkey, according to Chronic Diseases and Risk Factors Survey, CVD is also the number one cause of death with a 20 % rate of myocardial infarction^(6,7)^. All deaths from CVD are at 42 % in Turkey, and in the elderly population, this ratio increases to 54 %^(7)^. According to Turkish Adult Risk Factor Study (TEKHARF)-2017, there are 3·5 million people in Turkey suffering from CHD and, with the ageing population, there seems to be a 4 % increase annually^(8)^. Turkey has the highest coronary mortality rates in the 45–75-year-old population when compared with European countries^(8)^.

Dietary fat and cholesterol intake is considered as a risk factor for CVD, and behaviour changes towards decreasing the excess intake of fat and cholesterol seem to be beneficial for the prevention of CVD^(9)^. For the modification of dietary habits, it is crucial to assess the diet accurately and efficiently^(10)^. Commonly used dietary assessment tools are dietary records, dietary history, FFQ and 24-h dietary recall methods^(11)^. Majority of these methods take time to collect accurately and require software to analyse the fat and cholesterol intakes. Therefore, it could be very helpful to develop a rapid and accurate tool to measure far and cholesterol intake^(12)^. MEDFICTS (Meats, Eggs, Dairy, Fried foods, fat In baked goods, Convenience foods, fats added at the Table, Snacks) is a brief dietary assessment tool developed as part of the National Cholesterol Education Program Adult Treatment Panel guidelines^(12)^. It measures the adherence to Step 1 and Step 2 diets that are recommended for the prevention and treatment of CVD. Step 1 diet has <10 % of energy intake from saturated fat, <30 % of energy intake from total fat and <300 mg of dietary cholesterol per day. Step 2 diet has <7 % of energy from saturated fat, <200 mg dietary cholesterol per day and <30 % of energy intake from fat^(13,14)^.

In this study, we compared MEDFICTS questionnaire scores to total fat, SFA and cholesterol intake levels from 3-d food records in a group of university students without any CVD.

## Methods

### Subjects

Subjects were university students, recruited from several departments of Hacettepe University by trained dietitians in Ankara, Turkey. A total of 442 adults (249 females and 194 males) between the ages of 18 and 31 years participated in the study. The exclusion criteria were as follows: CVD, CHD, hypertension, hyperlipidaemia, CHD, cerebrovascular disease, previous heart surgery, any autoimmune disease, any chronic metabolic disease, any mental disease and pregnancy. All subjects were informed, and before testing, each participant gave written consent. The study was approved by the Institutional Review Board of Hacettepe University.

### Questionnaire

All subjects completed a short questionnaire, administered by a trained dietitian. Questions intended to collect data about sociodemographic information such as age, gender, education status, nutritional habits, meal frequency and physical activity habits. Anthropometric measurements like body weight (kg) and height (cm) were self-reported by the subjects.

### MEDFICTS

The MEDFICTS questionnaire^(12)^ composed of twenty food groups in nine sections: meats, eggs, dairy, frozen desserts, frying foods, in baked goods, convenience foods, table fats and snacks. Each food group in each section is questioned for weekly consumption frequency as rarely/never, 3 or less and 4 or less and for serving size as small, average and large for the meats group and as ≤1, 2 and ≥3 for the rest of the food groups. Points of weekly consumption frequency (0 = rarely/never, 3 = 3 or less, and 7 = 4 or more) are multiplied by serving size points (1 = small or ≤1, 2 = average or 2, and 3 = large or ≥3). For the lean meats group only, 6 points can be included if only there is a large size consumption. Subsequently, the sum is taken for all food items to calculate the final MEDFICTS score. A score of ≥70 is considered as high-fat diet, 40–70 is adherence to step 1 diet and <40 is adherence to step 2 diet^(12)^.

In this study, the MEDFICTS questionnaire was translated and adjusted to Turkish language and food culture using the Brislin method^(15)^. Fifteen native Turkish speaker trained dietitians translated the questionnaire from English to Turkish. Two native English speaker experts translated the form back to English. These translated forms were compared with the original form by an expert panel, and after deciding on necessary adjustment, the last version of the Turkish MEDFICTS was formed.

All participants repeated the MEDFICTS questionnaire 2 weeks after baseline administration to detect the test–retest reliability.

### 3-Day food records

All subjects were trained by a dietitian on how to keep a 3-d food record. Participants were asked to keep dietary records for three consecutive days including 1 weekend day. To minimise the recollection error, participants were asked to write down their food intake immediately after the meals. To minimise the portion size errors, participants were asked to record the food amounts using household measurements. After receiving the 3-d food records, a trained dietitian reviewed the records and interviewed the participant to clarify any unclear or missing information. Consecutively, all food records were reviewed by a supervisor dietitian for accuracy.

### Statistical analysis

Statistical analyses were performed using the computer programme SPSS 22.0 (Armonk, NY: IBM Corp). Kolmogorov–Smirnov test was used to assess normality. Student’s *t* test was used for mean comparison of two groups. One-way ANOVA was used for comparison of more than two group means. For *post hoc* analysis, Bonferroni correction was used with equal variances and Games–Howell test was used with unequal variances. Correlations among variables were tested with Pearson’s correlation coefficient. Nominal data were examined using *χ*^2^ test. Internal consistency of the MEDFICTS questionnaire was evaluated using Cronbach’s alpha. Test–retest reliability was evaluated by intra-class correlation coefficient. Receiver operating characteristics curve analysis was used to measure the sensitivity and specificity of MEDFICTS cut-off points. Data were presented as means ± SD and frequencies. Differences were considered significant at *P* < 0·05.

## Results

Average ages of the participants were 23·3 ± 1·48 years for females and 23·6 ± 1·70 years for males. Male participants had a slightly higher average BMI than females (23·6 ± 4·56 kg/m^2^ and 24·9 ± 4·16 kg/m^2^, respectively) (Supplementary data)

Energy and macronutrient ratios for energy of the participants from 3-d food records are listed in Table 1. Macronutrient ratios for energy did not show any difference between genders (*P* > 0·05). Total fat ratio was higher than the recommended level for both males and females (39·4 and 39·9 %, respectively). Furthermore, SFA intake ratios were also higher than the recommended level for both gender groups (13·5 % for females and 12·9 % for males). MEDFICTS total score and diet group distribution of the participants are listed in Table 1. Mean MEDCISTS score was 66·3 ± 27·24 points. Males had significantly higher MEDFICTS scores than females (70·1 ± 30·24 and 63·3 ± 24·29, respectively; *P* = 0·011). According to MEDFICTS scores, 41·1 % of the participants have a high-fat diet and only 15·4 % of the participants have a low-fat diet. Total energy, total fat, SFA and cholesterol intakes from 3-d food records within the different MEDFICTS diet groups significantly differed (Table 2, P < 0·001 for all).

**Table 1:** Energy and macronutrient contents from 3-d food records and MEDFICTS scores (*n* 442)

Energy and macronutrients ratio	Female (*n* 249)		Male (*n* 194)		All participants (*n* 442)		
	Mean	sd	Mean	sd	Mean	sd	P*
Energy (kcal)	2066	356·5	2442	528·4	2230	477·6	< 0·001
Protein (%)	15·7	3·36	15·5	3·63	15·6	3·47	> 0·05
Carbohydrate (%)	44·3	6·93	45·1	7·38	44·6	7·14	> 0·05
Total fat (%)	39·9	6·85	39·4	6·82	39·7	6·83	> 0·05
PUFA (%)	10·3	1·3	10·8	1·5	10·7	1·4	> 0·05
MUFA (%)	13·9	3·2	13·5	3·4	13·7	3·3	> 0·05
SFA (%)	13·5	0·9	12·9	1·2	13·3	1·1	> 0·05
MEDFICTS Data							
Total MEDFICTS Score	63·3	24·29	70·1	30·24	66·3	27·24	0·011
Diet Group Distribution (%)							
High-fat diet	34·9		44·3		41·1		0·028
Moderate-fat diet	49·8		40·2		45·1		0·028
Low-fat diet	15·3		15·5		15·4		> 0·05

MEDFICTS, Meats, Eggs, Dairy, Fried foods, fat In baked goods, Convenience foods, fats added at the Table, and Snacks.

*t test and *χ*^2^ test were used for analysis.

**Table 2:** Comparison of energy, fat and cholesterol intakes from 3-d food records to MEDFICTS diet groups (*n* 442)

3-D food record	High-fat diet (*n* 173)		Moderate-fat diet (*n* 202)		Low-fat diet (*n* 68)			
	Mean	sd	Mean	sd	Mean	sd	ANOVA F	P^*^
Total energy (kcal)	1874	523·1	1697	434·1	1462	326·8	20·8	< 0·001
Total fat (%)	40·1	7·66	39·7	67·05	31·8	5·29	15·4	< 0·001
SFA (%)	15·4	3·18	13·2	3·78	11·6	3·78	12·2	< 0·001
Cholesterol (mg)	293·3	10·71	265·3	8·27	220·7	11·31	8·5	< 0·001

MEDFICTS, Meats, Eggs, Dairy, Fried foods, fat In baked goods, Convenience foods, fats added at the Table, and Snacks.

* ANOVA test was used for analysis.

**Fig. 1:**
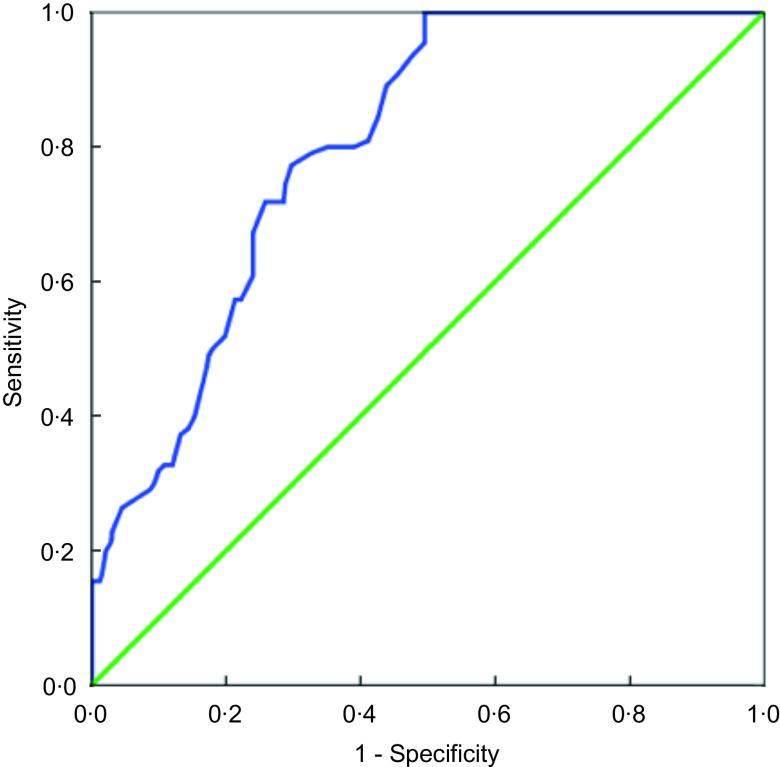
Receiver operating characteristic (ROC) curve showing the relationship between Adult Treatment Panel (ATP) guidelines and MEDFICTS groups at a cut-off point of 60. ROC AUC: 0·801, *P* < 0·001. Optimal cut-off point 60, 80% Sensitivity, 65% Specificity

Furthermore, receiver operating characteristics curve analysis demonstrated that a cut-off point of 60 showed 80 % sensitivity and 65 % specificity (Fig. 1). When receiver operating characteristic curve analysis was performed by gender, for males a cut-off point of 56 showed 84 % sensitivity and 78 % specificity and for females a cut-off point of 51 showed 72 % sensitivity and 56 % specificity (Supplementary data).

Test–retest reliability was tested with intra-class correlation coefficient, and intra-class correlation coefficient value was found as 0·891 (Supplementary data).

## Discussion

The prevalence of CVD in Turkey is estimated to increase to 4·2 million adults by 2025 and to 5·4 million by 2035, which is a 26 % and 58 % increase, respectively, compared with 2016^(16)^. According to AHA guidelines, a diet pattern increasing the intake of fish, nuts, whole grains, fruits and vegetables, and legumes, decreasing the intake of Na and cholesterol, replacing saturated fats with polyunsaturated and monounsaturated fats, avoiding trans fats and limiting the intake of processed meats, refined carbohydrates and sweetened drinks may reduce the risk of CVD^(17,18)^. Therefore, assessing dietary fat intake can be useful to implement an effective dietary intervention. In this study, we compared the MEDFICTS questionnaire with 3-d food records to evaluate the validity of MEDFICTS in Turkish population. Our data indicate that the MEDFICTS questionnaire is moderately accurate with 80 % sensitivity and 65 % specificity. However, sensitivity analysis did not demonstrate the recommended 40 points as an optimal cut-off point for Turkish population. Instead, at 60 cut-off point, maximum sensitivity (80 %) without significant loss in specificity (65 %) was achieved. Overall high sensitivity at the 60 cut-off point can be interpreted as MEDFICTS’ ability to identify people who are adherent to Step 1 Adult Treatment Panel guidelines; however, specificity seems to be decreasing with lower cut-off points; therefore, it may be ruling out high-fat dietary patterns. Low specificity with lower points may be due to the underestimation of fats and oils used in everyday cooking in Turkish population. Majority of Turkish cuisine recipes involve a fat or oil-based starter which is not reflected in MEDFICTS questionnaire.

In conclusion, although the MEDFICTS questionnaire may be used to identify people who are adherent to Step 1 Adult Treatment Panel guidelines, it needs to be adjusted to better estimate the overall dietary fat intake of Turkish population.

## Supplementary material

For supplementary material accompanying this paper visit https://doi.org/10.1017/S1368980021002299. click here to view supplementary material
