# Supplementary material for: Validation of MEDFICTS Dietary Assessment Questionnaire in Turkish Population
Source: Public Health Nutr. 2021 May 24;25(1):13–7. doi: 10.1017/S1368980021002299 (PMC8825974; doi:10.1017/S1368980021002299)
Supplement: Supplementary file 1 [file S1368980021002299sup001.docx]

**Supplementary Material**

**Table 1. Demographic and life-style characteristics of the subjects (n= 442).**

| **Characteristics** | **Female (n=249)** | **Male (n=194)** | **p*** |
| --- | --- | --- | --- |
| Age (yr) | 23.3 ± 1.48 | 23.6 ± 1.70 | >0.05 |
| BMI (kg/m^2^) | 23.6 ± 4.56 | 24.9 ± 4.16 | **0.004** |
| Underweight % | 6.8 | 4.6 | >0.05 |
| Normal % | 58.2 | 54.6 | >0.05 |
| Overweight % | 25.7 | 25.8 | >0.05 |
| Obese % | 9.2 | 14.9 | **0.045** |
| Smoking % (Y/N) | 12.0/88.0 | 29.5/70.5 | **<0.001** |
| Alcohol % (Y/N) | 14.1/85.9 | 30.1/69.9 | **<0.001** |
| Main meal (number) | 2.6 ± 0.51 | 2.7 ± 0.51 | >0.05 |
| Snacks (number) | 1.5 ± 0.93 | 1.5 ± 0.96 | >0.05 |
| Skipping meals % (Y/N) | 71.5/28.5 | 69.4/30.6 | >0.05 |
| Breakfast % | 52.9 | 70.2 | **0.045** |
| Lunch % | 38.7 | 20.2 | **0.040** |
| Dinner % | 8.4 | 9.5 | >0.05 |
| Regular exercise % (Y/N) | 30.9/69.1 | 48.1/51.8 | **<0.001** |

Abbreviations; BMI: Body Mass Index, Y/N: Yes/No.

*t test and Chi-square test were used for analysis.

**Table 2. Test-retest reliability of overall MEDFICTS (n=442).**

|  | **Cronbach’s α** | **Test-retest reliability ICC** | **Range of standardized factor loadings** | **p*** |
| --- | --- | --- | --- | --- |
| MEDFICTS | 0.891 | 0.891 | 0.869-0.910 | **<0.001** |

Abbreviations; ICC: Intraclass correlation coefficient, MEDFICTS: Meats, Eggs, Dairy, Fried foods, fat In baked goods, Convenience foods, fats added at the Table, and Snacks.

*Intraclass correlation coefficient was used for analysis.

**Figures**


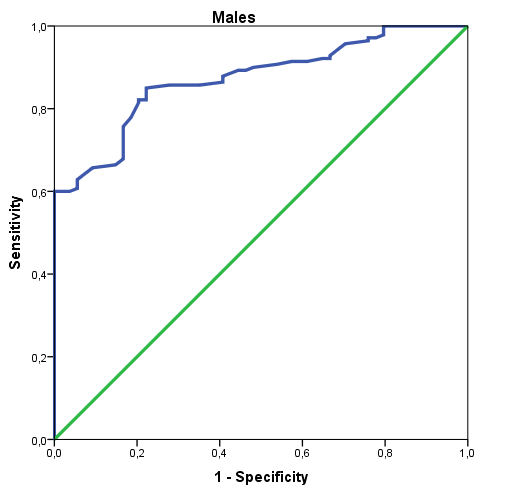


Figure 1. ROC curve showing the relationship between Adult Treatment Panel (ATP) guidelines and MEDFICTS groups at a cut-off point of 56 for males

ROC Area Under the Curve: 0.872, p<0.001

Optimal cut off point 56, %84 Sensitivity, %78 Specificity


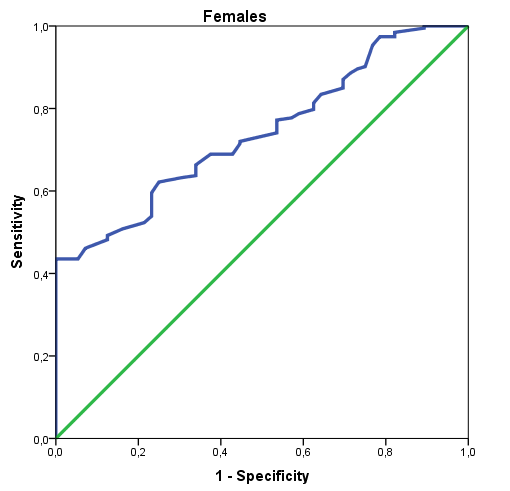


Figure 2. ROC curve showing the relationship between Adult Treatment Panel (ATP) guidelines and MEDFICTS groups at a cut-off point of 51 for females

ROC Area Under the Curve: 0.741, p<0.001

Optimal cut off point 51, %72 Sensitivity, %56 Specificity
